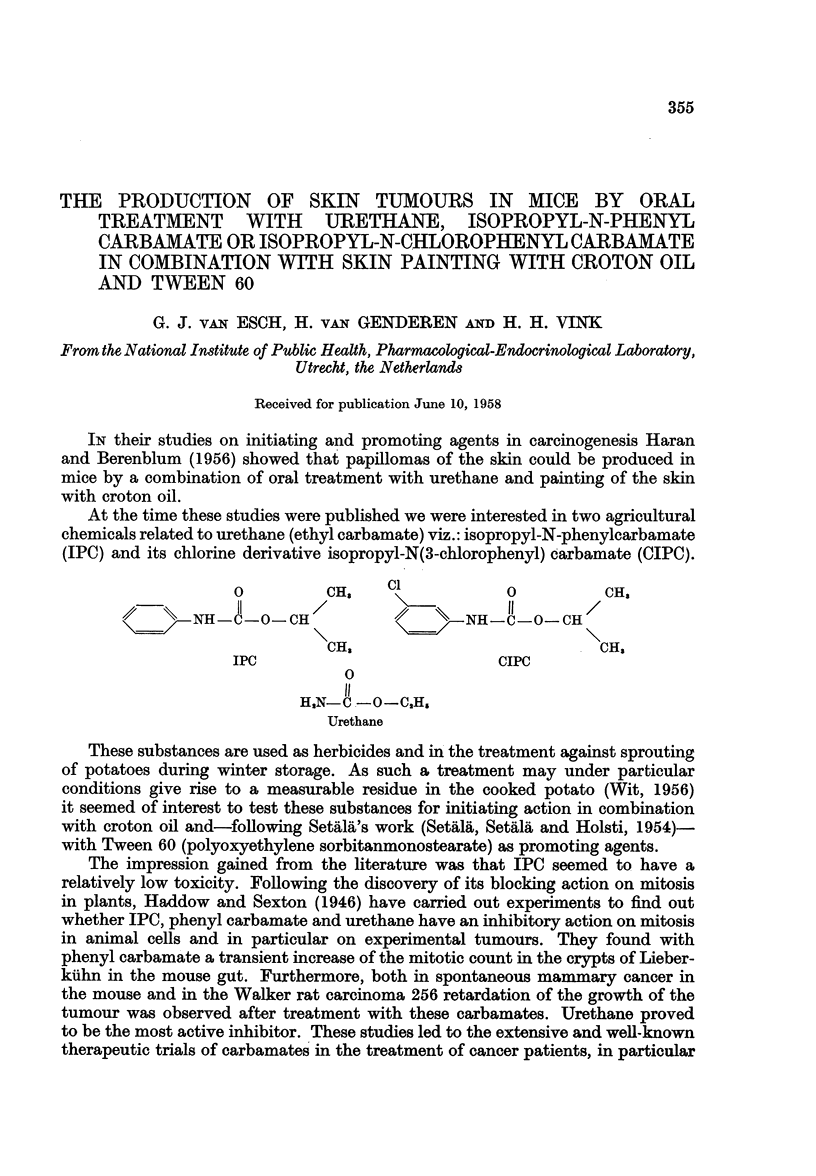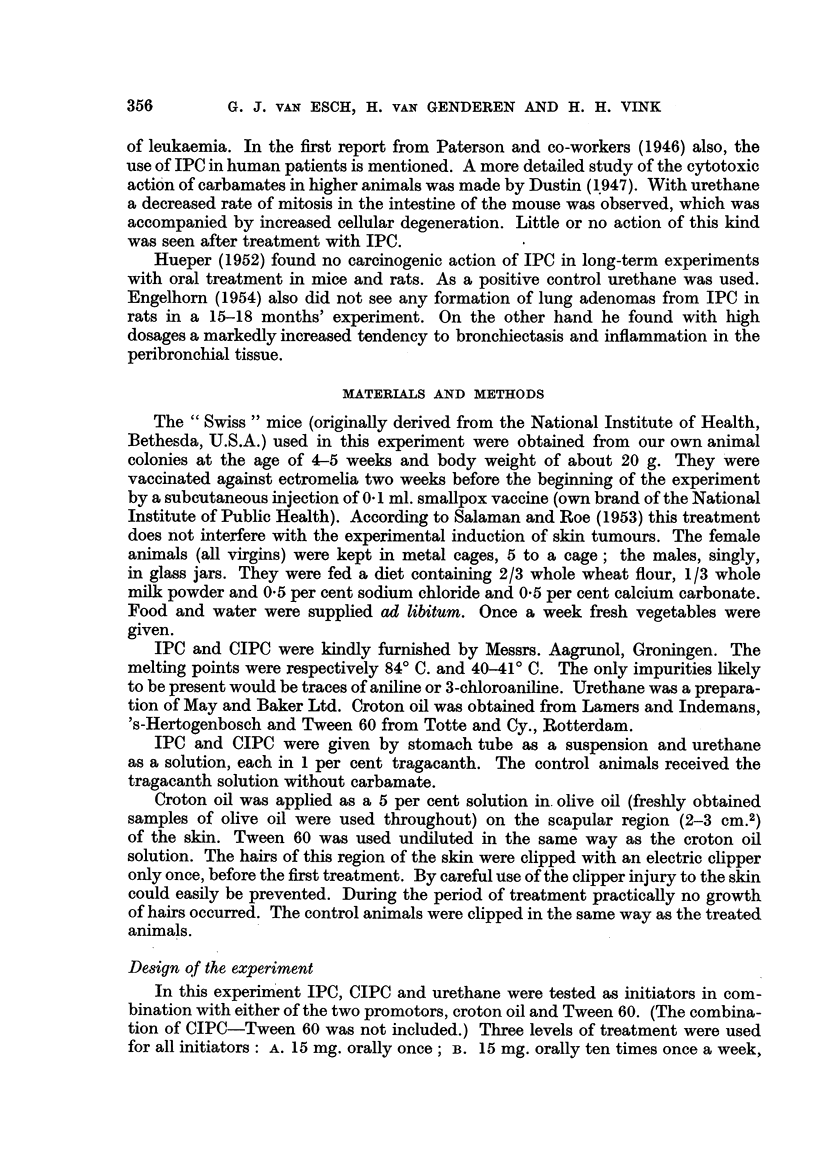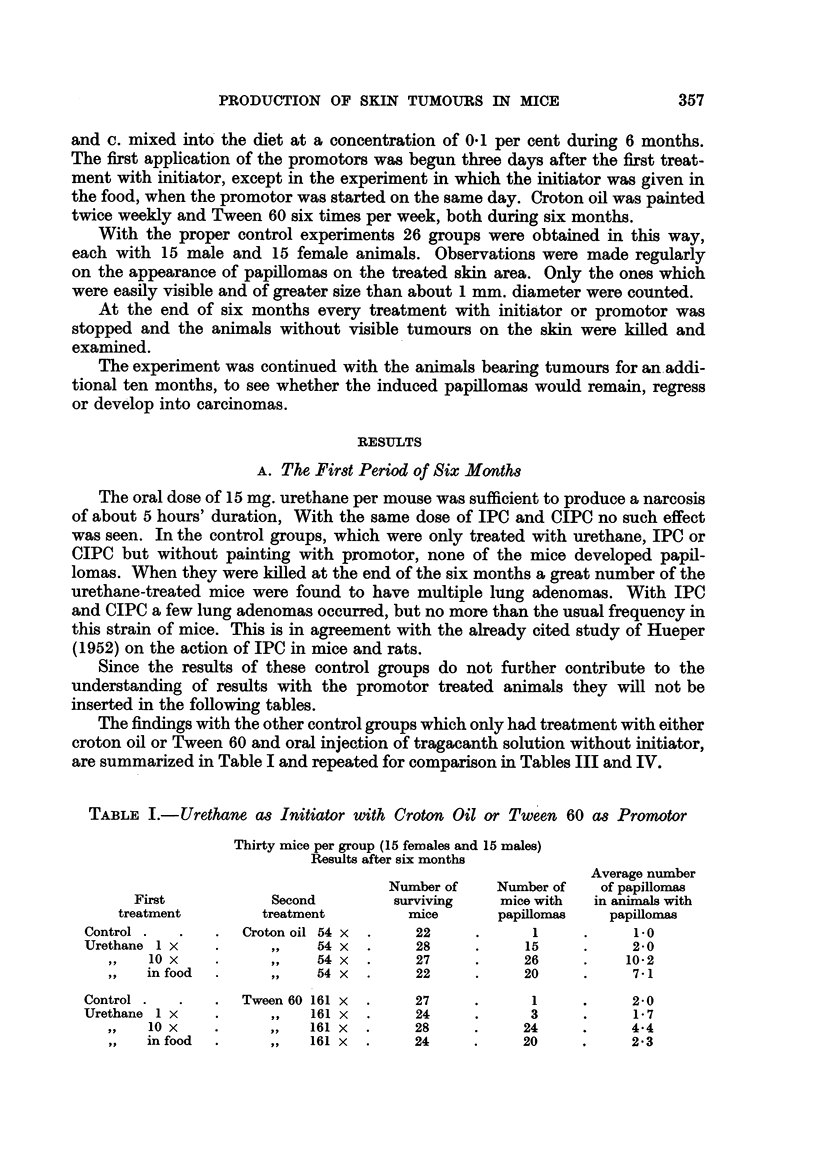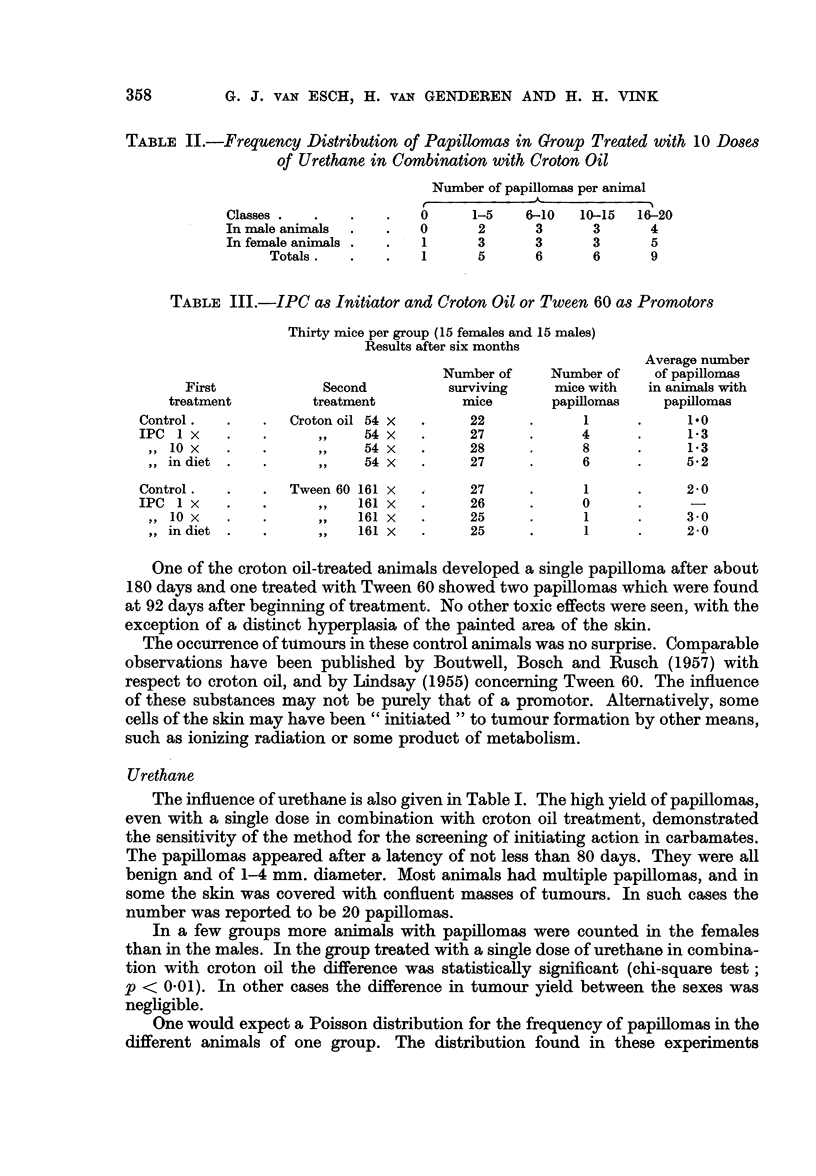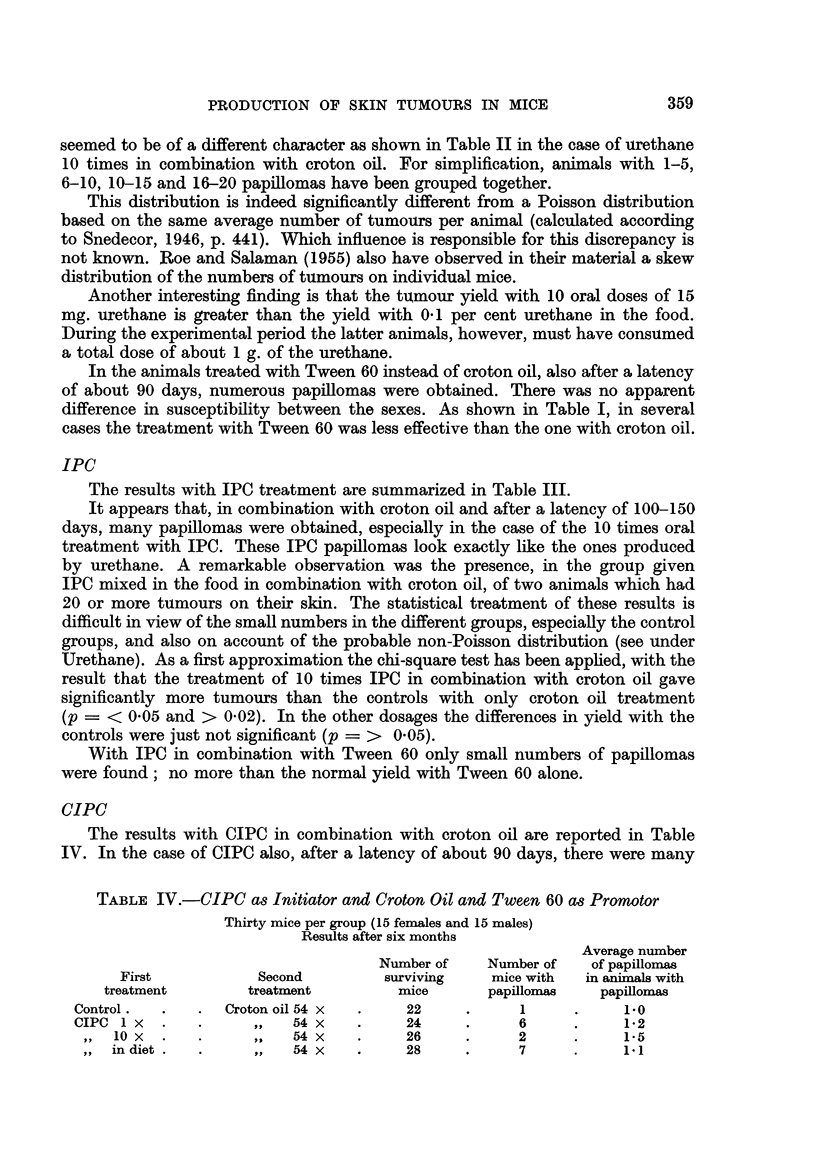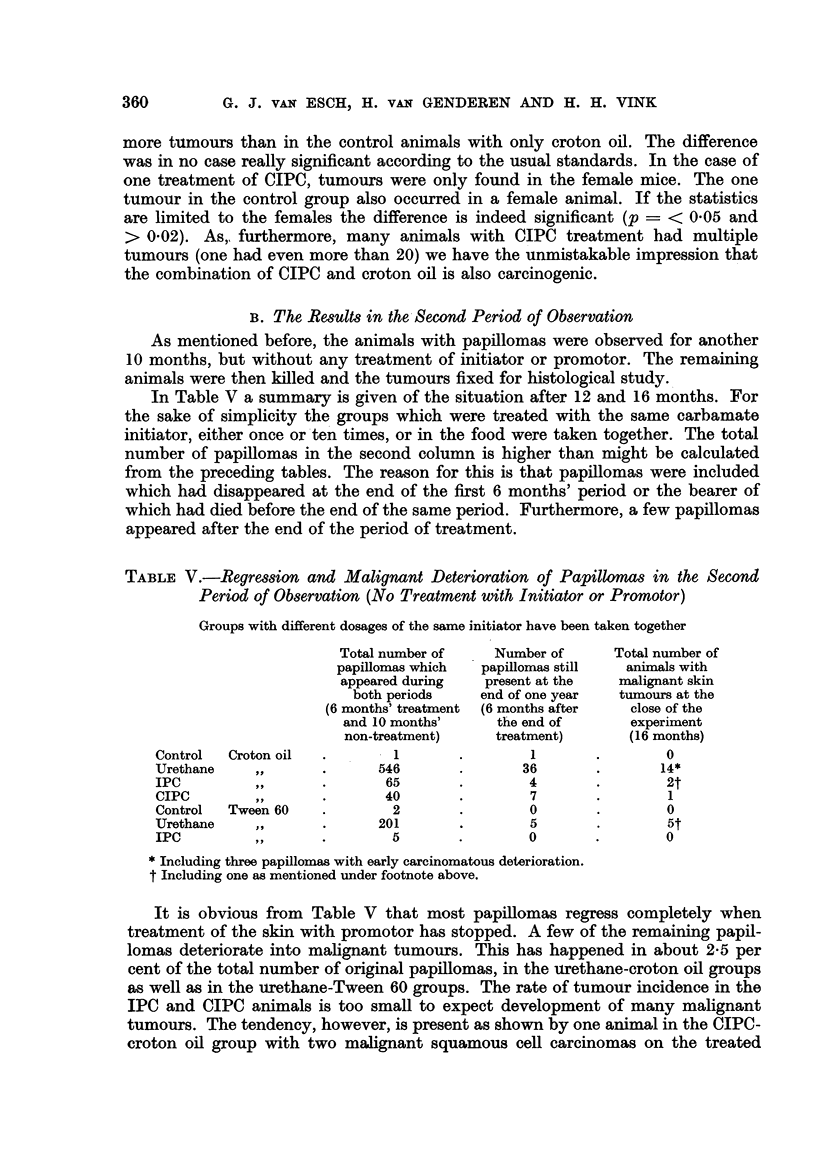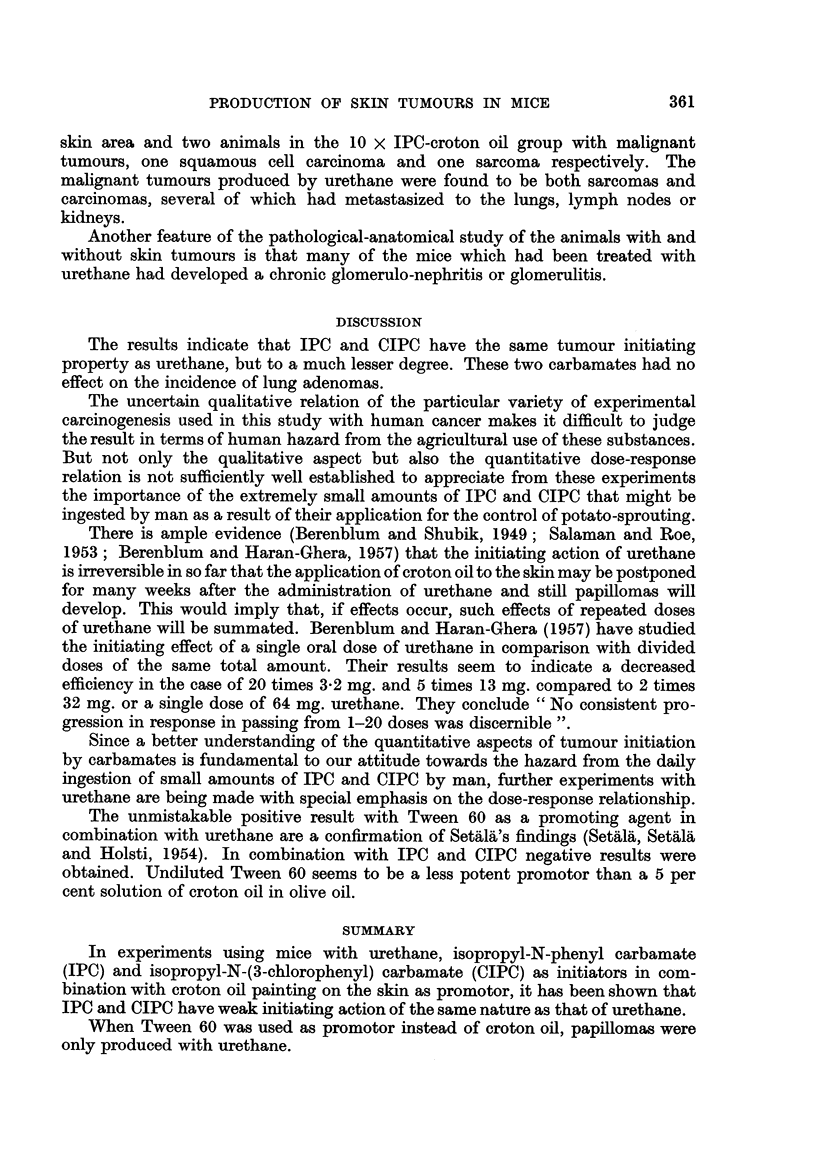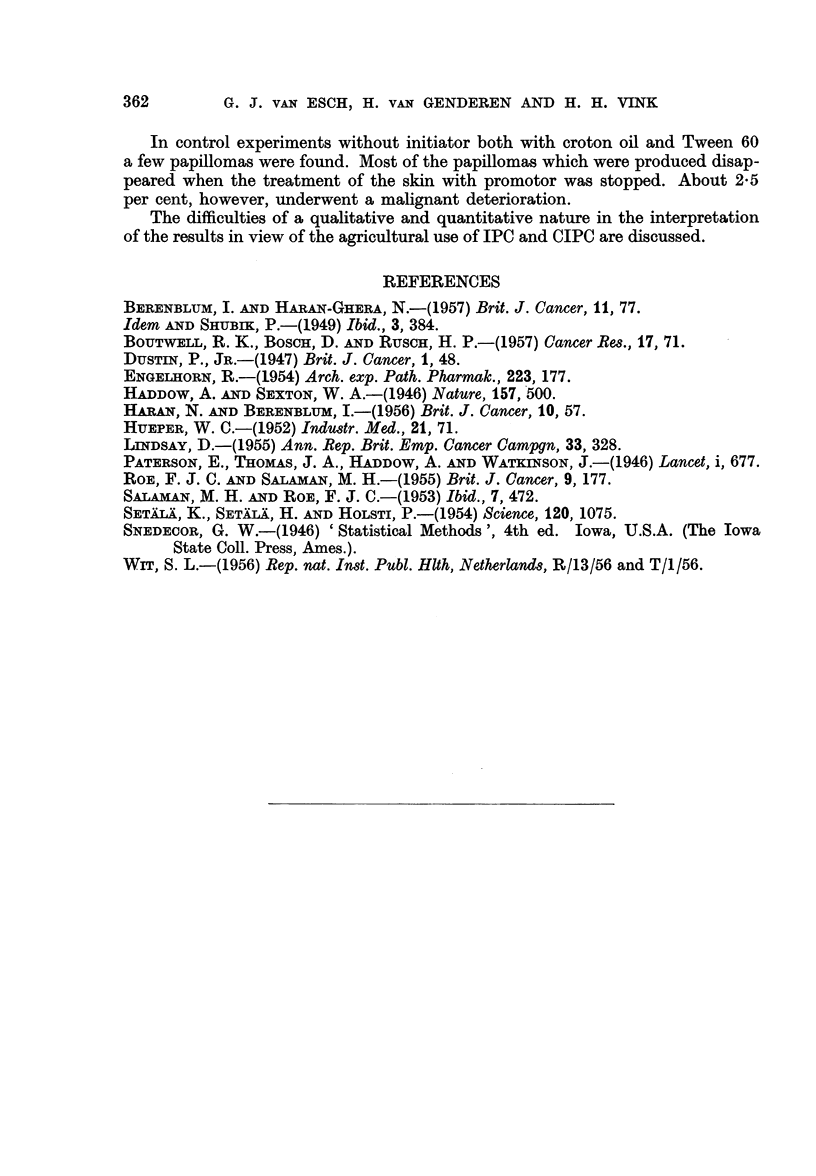# The Production of Skin Tumours in Mice by Oral Treatment with Urethane, Isopropyl-N-phenyl Carbamate or Isopropyl-N-chlorophenyl carbamate in Combination with Skin Painting with Croton Oil and Tween 60

**DOI:** 10.1038/bjc.1958.44

**Published:** 1958-09

**Authors:** G. J. van Esch, H. van Genderen, H. H. Vink


### THE PRODUCTION OF SKIN TUMOURS IN MICE BY ORAL TREATMENT WITH URETHANE, ISOPROPYL-N-PHENYL CARBAMATE OR ISOPROPYL-N-CHLOROPHENYL CARBAMATE IN COMBINATION WITH SKIN PAINTING WITH CROTON OIL AND TWEEN 60

G. J. VAN ESCH, H. VAN GENDEREN AND H. H. VINK

*From the National Institute of Public Health, Pharmacological-Endocrinological Laboratory, Utrecht, the Netherlands*

Received for publication June 10, 1958

IN their studies on initiating and promoting agents in carcinogenesis Haran and Berenblum (1956) showed that papillomas of the skin could be produced in mice by a combination of oral treatment with urethane and painting of the skin with croton oil.

At the time these studies were published we were interested in two agricultural chemicals related to urethane (ethyl carbamate) viz.: isopropyl-N-phenylcarbamate (IPC) and its chlorine derivative isopropyl-N(3-chlorophenyl) carbamate (CIPC).

IPC

CIPC

Urethane

These substances are used as herbicides and in the treatment against sprouting of potatoes during winter storage. As such a treatment may under particular conditions give rise to a measurable residue in the cooked potato (Wit, 1956) it seemed of interest to test these substances for initiating action in combination with croton oil and—following Setälä's work (Setälä, Setälä and Holsti, 1954)—with Tween 60 (polyoxyethylene sorbitanmonostearate) as promoting agents.

The impression gained from the literature was that IPC seemed to have a relatively low toxicity. Following the discovery of its blocking action on mitosis in plants, Haddow and Sexton (1946) have carried out experiments to find out whether IPC, phenyl carbamate and urethane have an inhibitory action on mitosis in animal cells and in particular on experimental tumours. They found with phenyl carbamate a transient increase of the mitotic count in the crypts of Lieberkühn in the mouse gut. Furthermore, both in spontaneous mammary cancer in the mouse and in the Walker rat carcinoma 256 retardation of the growth of the tumour was observed after treatment with these carbamates. Urethane proved to be the most active inhibitor. These studies led to the extensive and well-known therapeutic trials of carbamates in the treatment of cancer patients, in particular of leukaemia. In the first report from Paterson and co-workers (1946) also, the use of IPC in human patients is mentioned. A more detailed study of the cytotoxic action of carbamates in higher animals was made by Dustin (1947). With urethane a decreased rate of mitosis in the intestine of the mouse was observed, which was accompanied by increased cellular degeneration. Little or no action of this kind was seen after treatment with IPC.

Hueper (1952) found no carcinogenic action of IPC in long-term experiments with oral treatment in mice and rats. As a positive control urethane was used. Engelhorn (1954) also did not see any formation of lung adenomas from IPC in rats in a 15–18 months' experiment. On the other hand he found with high dosages a markedly increased tendency to bronchiectasis and inflammation in the peribronchial tissue.

### MATERIALS AND METHODS

The " Swiss " mice (originally derived from the National Institute of Health, Bethesda, U.S.A.) used in this experiment were obtained from our own animal colonies at the age of 4–5 weeks and body weight of about 20 g. They were vaccinated against ectromelia two weeks before the beginning of the experiment by a subcutaneous injection of 0·1 ml. smallpox vaccine (own brand of the National Institute of Public Health). According to Salaman and Roe (1953) this treatment does not interfere with the experimental induction of skin tumours. The female animals (all virgins) were kept in metal cages, 5 to a cage ; the males, singly, in glass jars. They were fed a diet containing 2/3 whole wheat flour, 1/3 whole milk powder and 0·5 per cent sodium chloride and 0·5 per cent calcium carbonate. Food and water were supplied *ad libitum*. Once a week fresh vegetables were given.

IPC and CIPC were kindly furnished by Messrs. Aagrunol, Groningen. The melting points were respectively 84° C. and 40–41° C. The only impurities likely to be present would be traces of aniline or 3-chloroaniline. Urethane was a preparation of May and Baker Ltd. Croton oil was obtained from Lamers and Indemans, 's-Hertogenbosch and Tween 60 from Totte and Cy., Rotterdam.

IPC and CIPC were given by stomach tube as a suspension and urethane as a solution, each in 1 per cent tragacanth. The control animals received the tragacanth solution without carbamate.

Croton oil was applied as a 5 per cent solution in olive oil (freshly obtained samples of olive oil were used throughout) on the scapular region (2–3 $cm.^2$) of the skin. Tween 60 was used undiluted in the same way as the croton oil solution. The hairs of this region of the skin were clipped with an electric clipper only once, before the first treatment. By careful use of the clipper injury to the skin could easily be prevented. During the period of treatment practically no growth of hairs occurred. The control animals were clipped in the same way as the treated animals.

### *Design of the experiment*

In this experiment IPC, CIPC and urethane were tested as initiators in combination with either of the two promotors, croton oil and Tween 60. (The combination of CIPC—Tween 60 was not included.) Three levels of treatment were used for all initiators : A. 15 mg. orally once ; B. 15 mg. orally ten times once a week, and C. mixed into the diet at a concentration of 0·1 per cent during 6 months. The first application of the promotors was begun three days after the first treatment with initiator, except in the experiment in which the initiator was given in the food, when the promotor was started on the same day. Croton oil was painted twice weekly and Tween 60 six times per week, both during six months.

With the proper control experiments 26 groups were obtained in this way, each with 15 male and 15 female animals. Observations were made regularly on the appearance of papillomas on the treated skin area. Only the ones which were easily visible and of greater size than about 1 mm. diameter were counted.

At the end of six months every treatment with initiator or promotor was stopped and the animals without visible tumours on the skin were killed and examined.

The experiment was continued with the animals bearing tumours for an additional ten months, to see whether the induced papillomas would remain, regress or develop into carcinomas.

### RESULTS

### A. *The First Period of Six Months*

The oral dose of 15 mg. urethane per mouse was sufficient to produce a narcosis of about 5 hours' duration, With the same dose of IPC and CIPC no such effect was seen. In the control groups, which were only treated with urethane, IPC or CIPC but without painting with promotor, none of the mice developed papillomas. When they were killed at the end of the six months a great number of the urethane-treated mice were found to have multiple lung adenomas. With IPC and CIPC a few lung adenomas occurred, but no more than the usual frequency in this strain of mice. This is in agreement with the already cited study of Hueper (1952) on the action of IPC in mice and rats.

Since the results of these control groups do not further contribute to the understanding of results with the promotor treated animals they will not be inserted in the following tables.

The findings with the other control groups which only had treatment with either croton oil or Tween 60 and oral injection of tragacanth solution without initiator, are summarized in Table I and repeated for comparison in Tables III and IV.

TABLE I.—*Urethane as Initiator with Croton Oil or Tween 60 as Promotor*

Thirty mice per group (15 females and 15 males)
Results after six months

| First treatment | Second treatment | Number of surviving mice | Number of mice with papillomas | Average number of papillomas in animals with papillomas |
|---|---|---|---|---|
| Control | Croton oil 54 × | 22 | 1 | 1·0 |
| Urethane 1 × | ,, 54 × | 28 | 15 | 2·0 |
| ,, 10 × | ,, 54 × | 27 | 26 | 10·2 |
| ,, in food | ,, 54 × | 22 | 20 | 7·1 |
| Control | Tween 60 161 × | 27 | 1 | 2·0 |
| Urethane 1 × | ,, 161 × | 24 | 3 | 1·7 |
| ,, 10 × | ,, 161 × | 28 | 24 | 4·4 |
| ,, in food | ,, 161 × | 24 | 20 | 2·3 |

TABLE II.—*Frequency Distribution of Papillomas in Group Treated with* 10 *Doses of Urethane in Combination with Croton Oil*

| | Number of papillomas per animal | | | | |
|---|---|---|---|---|---|
| Classes | 0 | 1–5 | 6–10 | 10–15 | 16–20 |
| In male animals | 0 | 2 | 3 | 3 | 4 |
| In female animals | 1 | 3 | 3 | 3 | 5 |
| Totals | 1 | 5 | 6 | 6 | 9 |

TABLE III.—*IPC as Initiator and Croton Oil or Tween* 60 *as Promotors*

Thirty mice per group (15 females and 15 males)
Results after six months

| First treatment | Second treatment | Number of surviving mice | Number of mice with papillomas | Average number of papillomas in animals with papillomas |
|---|---|---|---|---|
| Control | Croton oil 54 × | 22 | 1 | 1·0 |
| IPC 1 × | ,, 54 × | 27 | 4 | 1·3 |
| ,, 10 × | ,, 54 × | 28 | 8 | 1·3 |
| ,, in diet | ,, 54 × | 27 | 6 | 5·2 |
| Control | Tween 60 161 × | 27 | 1 | 2·0 |
| IPC 1 × | ,, 161 × | 26 | 0 | — |
| ,, 10 × | ,, 161 × | 25 | 1 | 3·0 |
| ,, in diet | ,, 161 × | 25 | 1 | 2·0 |

One of the croton oil-treated animals developed a single papilloma after about 180 days and one treated with Tween 60 showed two papillomas which were found at 92 days after beginning of treatment. No other toxic effects were seen, with the exception of a distinct hyperplasia of the painted area of the skin.

The occurrence of tumours in these control animals was no surprise. Comparable observations have been published by Boutwell, Bosch and Rusch (1957) with respect to croton oil, and by Lindsay (1955) concerning Tween 60. The influence of these substances may not be purely that of a promotor. Alternatively, some cells of the skin may have been " initiated " to tumour formation by other means, such as ionizing radiation or some product of metabolism.

*Urethane*

The influence of urethane is also given in Table I. The high yield of papillomas, even with a single dose in combination with croton oil treatment, demonstrated the sensitivity of the method for the screening of initiating action in carbamates. The papillomas appeared after a latency of not less than 80 days. They were all benign and of 1–4 mm. diameter. Most animals had multiple papillomas, and in some the skin was covered with confluent masses of tumours. In such cases the number was reported to be 20 papillomas.

In a few groups more animals with papillomas were counted in the females than in the males. In the group treated with a single dose of urethane in combination with croton oil the difference was statistically significant (chi-square test ; $p < 0{\cdot}01$). In other cases the difference in tumour yield between the sexes was negligible.

One would expect a Poisson distribution for the frequency of papillomas in the different animals of one group. The distribution found in these experiments seemed to be of a different character as shown in Table II in the case of urethane 10 times in combination with croton oil. For simplification, animals with 1–5, 6–10, 10–15 and 16–20 papillomas have been grouped together.

This distribution is indeed significantly different from a Poisson distribution based on the same average number of tumours per animal (calculated according to Snedecor, 1946, p. 441). Which influence is responsible for this discrepancy is not known. Roe and Salaman (1955) also have observed in their material a skew distribution of the numbers of tumours on individual mice.

Another interesting finding is that the tumour yield with 10 oral doses of 15 mg. urethane is greater than the yield with 0·1 per cent urethane in the food. During the experimental period the latter animals, however, must have consumed a total dose of about 1 g. of the urethane.

In the animals treated with Tween 60 instead of croton oil, also after a latency of about 90 days, numerous papillomas were obtained. There was no apparent difference in susceptibility between the sexes. As shown in Table I, in several cases the treatment with Tween 60 was less effective than the one with croton oil.

*IPC*

The results with IPC treatment are summarized in Table III.

It appears that, in combination with croton oil and after a latency of 100–150 days, many papillomas were obtained, especially in the case of the 10 times oral treatment with IPC. These IPC papillomas look exactly like the ones produced by urethane. A remarkable observation was the presence, in the group given IPC mixed in the food in combination with croton oil, of two animals which had 20 or more tumours on their skin. The statistical treatment of these results is difficult in view of the small numbers in the different groups, especially the control groups, and also on account of the probable non-Poisson distribution (see under Urethane). As a first approximation the chi-square test has been applied, with the result that the treatment of 10 times IPC in combination with croton oil gave significantly more tumours than the controls with only croton oil treatment ($p = < 0{\cdot}05$ and $> 0{\cdot}02$). In the other dosages the differences in yield with the controls were just not significant ($p = > 0{\cdot}05$).

With IPC in combination with Tween 60 only small numbers of papillomas were found ; no more than the normal yield with Tween 60 alone.

*CIPC*

The results with CIPC in combination with croton oil are reported in Table IV. In the case of CIPC also, after a latency of about 90 days, there were many

TABLE IV.—*CIPC as Initiator and Croton Oil and Tween* 60 *as Promotor*

Thirty mice per group (15 females and 15 males)
Results after six months

| First treatment | Second treatment | Number of surviving mice | Number of mice with papillomas | Average number of papillomas in animals with papillomas |
|---|---|---|---|---|
| Control | Croton oil 54 × | 22 | 1 | 1·0 |
| CIPC 1 × | ,, 54 × | 24 | 6 | 1·2 |
| ,, 10 × | ,, 54 × | 26 | 2 | 1·5 |
| ,, in diet | ,, 54 × | 28 | 7 | 1·1 |

more tumours than in the control animals with only croton oil. The difference was in no case really significant according to the usual standards. In the case of one treatment of CIPC, tumours were only found in the female mice. The one tumour in the control group also occurred in a female animal. If the statistics are limited to the females the difference is indeed significant ($p = < 0{\cdot}05$ and $> 0{\cdot}02$). As, furthermore, many animals with CIPC treatment had multiple tumours (one had even more than 20) we have the unmistakable impression that the combination of CIPC and croton oil is also carcinogenic.

### B. *The Results in the Second Period of Observation*

As mentioned before, the animals with papillomas were observed for another 10 months, but without any treatment of initiator or promotor. The remaining animals were then killed and the tumours fixed for histological study.

In Table V a summary is given of the situation after 12 and 16 months. For the sake of simplicity the groups which were treated with the same carbamate initiator, either once or ten times, or in the food were taken together. The total number of papillomas in the second column is higher than might be calculated from the preceding tables. The reason for this is that papillomas were included which had disappeared at the end of the first 6 months' period or the bearer of which had died before the end of the same period. Furthermore, a few papillomas appeared after the end of the period of treatment.

TABLE V.—*Regression and Malignant Deterioration of Papillomas in the Second Period of Observation (No Treatment with Initiator or Promotor)*

Groups with different dosages of the same initiator have been taken together

| | | Total number of papillomas which appeared during both periods (6 months' treatment and 10 months' non-treatment) | Number of papillomas still present at the end of one year (6 months after the end of treatment) | Total number of animals with malignant skin tumours at the close of the experiment (16 months) |
|---|---|---|---|---|
| Control | Croton oil | 1 | 1 | 0 |
| Urethane | ,, | 546 | 36 | 14* |
| IPC | ,, | 65 | 4 | 2† |
| CIPC | ,, | 40 | 7 | 1 |
| Control | Tween 60 | 2 | 0 | 0 |
| Urethane | ,, | 201 | 5 | 5† |
| IPC | ,, | 5 | 0 | 0 |

* Including three papillomas with early carcinomatous deterioration.
† Including one as mentioned under footnote above.

It is obvious from Table V that most papillomas regress completely when treatment of the skin with promotor has stopped. A few of the remaining papillomas deteriorate into malignant tumours. This has happened in about 2·5 per cent of the total number of original papillomas, in the urethane-croton oil groups as well as in the urethane-Tween 60 groups. The rate of tumour incidence in the IPC and CIPC animals is too small to expect development of many malignant tumours. The tendency, however, is present as shown by one animal in the CIPC-croton oil group with two malignant squamous cell carcinomas on the treated skin area and two animals in the 10 × IPC-croton oil group with malignant tumours, one squamous cell carcinoma and one sarcoma respectively. The malignant tumours produced by urethane were found to be both sarcomas and carcinomas, several of which had metastasized to the lungs, lymph nodes or kidneys.

Another feature of the pathological-anatomical study of the animals with and without skin tumours is that many of the mice which had been treated with urethane had developed a chronic glomerulo-nephritis or glomerulitis.

### DISCUSSION

The results indicate that IPC and CIPC have the same tumour initiating property as urethane, but to a much lesser degree. These two carbamates had no effect on the incidence of lung adenomas.

The uncertain qualitative relation of the particular variety of experimental carcinogenesis used in this study with human cancer makes it difficult to judge the result in terms of human hazard from the agricultural use of these substances. But not only the qualitative aspect but also the quantitative dose-response relation is not sufficiently well established to appreciate from these experiments the importance of the extremely small amounts of IPC and CIPC that might be ingested by man as a result of their application for the control of potato-sprouting.

There is ample evidence (Berenblum and Shubik, 1949 ; Salaman and Roe, 1953 ; Berenblum and Haran-Ghera, 1957) that the initiating action of urethane is irreversible in so far that the application of croton oil to the skin may be postponed for many weeks after the administration of urethane and still papillomas will develop. This would imply that, if effects occur, such effects of repeated doses of urethane will be summated. Berenblum and Haran-Ghera (1957) have studied the initiating effect of a single oral dose of urethane in comparison with divided doses of the same total amount. Their results seem to indicate a decreased efficiency in the case of 20 times 3·2 mg. and 5 times 13 mg. compared to 2 times 32 mg. or a single dose of 64 mg. urethane. They conclude " No consistent progression in response in passing from 1–20 doses was discernible ".

Since a better understanding of the quantitative aspects of tumour initiation by carbamates is fundamental to our attitude towards the hazard from the daily ingestion of small amounts of IPC and CIPC by man, further experiments with urethane are being made with special emphasis on the dose-response relationship.

The unmistakable positive result with Tween 60 as a promoting agent in combination with urethane are a confirmation of Setälä's findings (Setälä, Setälä and Holsti, 1954). In combination with IPC and CIPC negative results were obtained. Undiluted Tween 60 seems to be a less potent promotor than a 5 per cent solution of croton oil in olive oil.

### SUMMARY

In experiments using mice with urethane, isopropyl-N-phenyl carbamate (IPC) and isopropyl-N-(3-chlorophenyl) carbamate (CIPC) as initiators in combination with croton oil painting on the skin as promotor, it has been shown that IPC and CIPC have weak initiating action of the same nature as that of urethane.

When Tween 60 was used as promotor instead of croton oil, papillomas were only produced with urethane.

In control experiments without initiator both with croton oil and Tween 60 a few papillomas were found. Most of the papillomas which were produced disappeared when the treatment of the skin with promotor was stopped. About 2·5 per cent, however, underwent a malignant deterioration.

The difficulties of a qualitative and quantitative nature in the interpretation of the results in view of the agricultural use of IPC and CIPC are discussed.

### REFERENCES

BERENBLUM, I. AND HARAN-GHERA, N.—(1957) *Brit. J. Cancer*, **11**, 77.
*Idem* AND SHUBIK, P.—(1949) *Ibid.*, **3**, 384.
BOUTWELL, R. K., BOSCH, D. AND RUSCH, H. P.—(1957) *Cancer Res.*, **17**, 71.
DUSTIN, P., JR.—(1947) *Brit. J. Cancer*, **1**, 48.
ENGELHORN, R.—(1954) *Arch. exp. Path. Pharmak.*, **223**, 177.
HADDOW, A. AND SEXTON, W. A.—(1946) *Nature*, **157**, 500.
HARAN, N. AND BERENBLUM, I.—(1956) *Brit. J. Cancer*, **10**, 57.
HUEPER, W. C.—(1952) *Industr. Med.*, **21**, 71.
LINDSAY, D.—(1955) *Ann. Rep. Brit. Emp. Cancer Campgn*, **33**, 328.
PATERSON, E., THOMAS, J. A., HADDOW, A. AND WATKINSON, J.—(1946) *Lancet*, i, 677.
ROE, F. J. C. AND SALAMAN, M. H.—(1955) *Brit. J. Cancer*, **9**, 177.
SALAMAN, M. H. AND ROE, F. J. C.—(1953) *Ibid.*, **7**, 472.
SETÄLÄ, K., SETÄLÄ, H. AND HOLSTI, P.—(1954) *Science*, **120**, 1075.
SNEDECOR, G. W.—(1946) 'Statistical Methods', 4th ed. Iowa, U.S.A. (The Iowa State Coll. Press, Ames.).
WIT, S. L.—(1956) *Rep. nat. Inst. Publ. Hlth, Netherlands*, R/13/56 and T/1/56.